# Apoptosis in Postmortal Tissues of Goat Spinal Cords and Survival of Resident Neural Progenitors

**DOI:** 10.3390/ijms25094683

**Published:** 2024-04-25

**Authors:** Andrey Mikhailov, Yoshiyuki Sankai

**Affiliations:** 1 Center for Cybernics Research, University of Tsukuba, Tsukuba 305-8573, Japan; 2 Faculty of Engineering, Information and Systems, University of Tsukuba, Tsukuba 305-8573, Japan; sec@golem.iit.tsukuba.ac.jp

**Keywords:** neuronal progenitor, cadaveric stem cells, apoptosis, spinal cord, caspase, GD2

## Abstract

Growing demand for therapeutic tissue repair recurrently focusses scientists’ attention on critical assessment of postmortal collection of live cells, especially stem cells. Our study aimed to assess the survival of neuronal progenitors in postmortal spinal cord and their differentiation potential. Postmortal samples of spinal cords were obtained from human-sized animals (goats) at 6, 12, 24, 36, and 54 h after slaughter. Samples were studied by immunohistology, differentiation assay, Western blot and flow cytometry for the presence and location of GD2-positive neural progenitors and their susceptibility to cell death. TUNEL staining of the goat spinal cord samples over 6–54 h postmortem revealed no difference in the number of positive cells per cross-section. Many TUNEL-positive cells were located in the gray commissure around the central canal of the spinal cord; no increase in TUNEL-positive cells was recorded in either posterior or anterior horns of the gray matter where many GD2-positive neural progenitors can be found. The active caspase 3 amount as measured by Western blot at the same intervals was moderately increasing over time. Neuronal cells were enriched by magnetic separation with antibodies against CD24; among them, the GD2-positive neural progenitor subpopulation did not overlap with apoptotic cells having high pan-caspase activity. Apoptotic cell death events are relatively rare in postmortal spinal cords and are not increased in areas of the neural progenitor cell’s location, within measured postmortal intervals, or among the CD24/GD2-positive cells. Data from our study suggest postmortal spinal cords as a valuable source for harvesting highly viable allogenic neural progenitor cells.

## 1. Introduction

Growing demand for therapeutic tissue repair and the problems with cryopreservation of whole organs recurrently focus scientists’ attention on the critical assessment of postmortal collection of live cells, especially stem cells. Cadaveric stem cells (CSCs), mesenchymal, adipose-delivered, neural, retinal, and hematopoietic, were successfully recovered and evaluated from different animal sources (reviewed in [1] and [2]). The extraction of the stem cells from cadavers demonstrates the ability of these cells to survive adverse conditions, including long-time cryopreservation [3]. Published therapeutic interventions using cadaveric stem cells include liver repopulation with hepatocytes derived up to 27 h postmortem in mice [4] and a recent, successful treatment of a patient having large severe burns with cadaveric bone marrow mesenchymal stem cells [5].

Cells in refrigerated cadavers can survive surprisingly long time. For example, the recovery of fibroblast-like cells from refrigerated goat skin is possible up to 41 days [6] postmortem, while the outgrowth of cells from bovine explants can withstand up to 49 days of postmortem storage [7]. Regarding stem cells, the shorter intervals are usually evaluated, like equine ligaments mesenchymal stem cells (MSCs) collected up to 72 h postmortem [8] or human MSC-like cells seen for up to 69 h [9]. There are no ideal animal models resembling all the aspects of human anatomy, physiology, and genetics. Large, domesticated animals come as models of choice when surgical techniques or large-scale cell harvesting is employed. We chose goats as our model due to their availability, anatomical similarities to humans, long lifespan, and the growing interest in using this model for stem cell research [10]. Even widely employed MSC-based products suffer from a lack of standardization among tissue sources and species [11]; regarding neural stem/progenitor cells, such concerns are also of crucial importance.

Central neural system repairs are especially challenging and costly, whereas the inability to complete clinical recovery has devastating consequences. Mechanisms and prospects for use of neural transplants for brain repair are extensively reviewed [12], and growing data suggest that postmortem human brain tissue might be an important source of neural progenitor cells useful for analysis of neural differentiation and for transplantation studies [13]. Albeit wide acceptance of transplantation practices, the concept of “brain death” is still under heavy criticism from some scholars of ethics [14]. “Biological death” when respiration and circulation has ceased for hours is much a clearer concept from an ethical point of view.

There are gaps between the knowledge of neuronal cell survival during organism development, the behavior of those cells in cell cultures under stress and treatments, and mostly unexplored changes in neural tissue after the organism dies. Our study is aimed at the assessment of feasibility for harvesting donor cadaveric neural stem cells from biologically dead mammalian organisms of human size and explores the perspective of a neural stem cell repository from spinal cords. Morphological features of the healthy human cadaveric spinal cord [15] are not much different from those of a goat spinal cord, so these human-sized animals were a rational selection for our study.

## 2. Results

### 2.1. Histological Analysis

**Figure 1:**
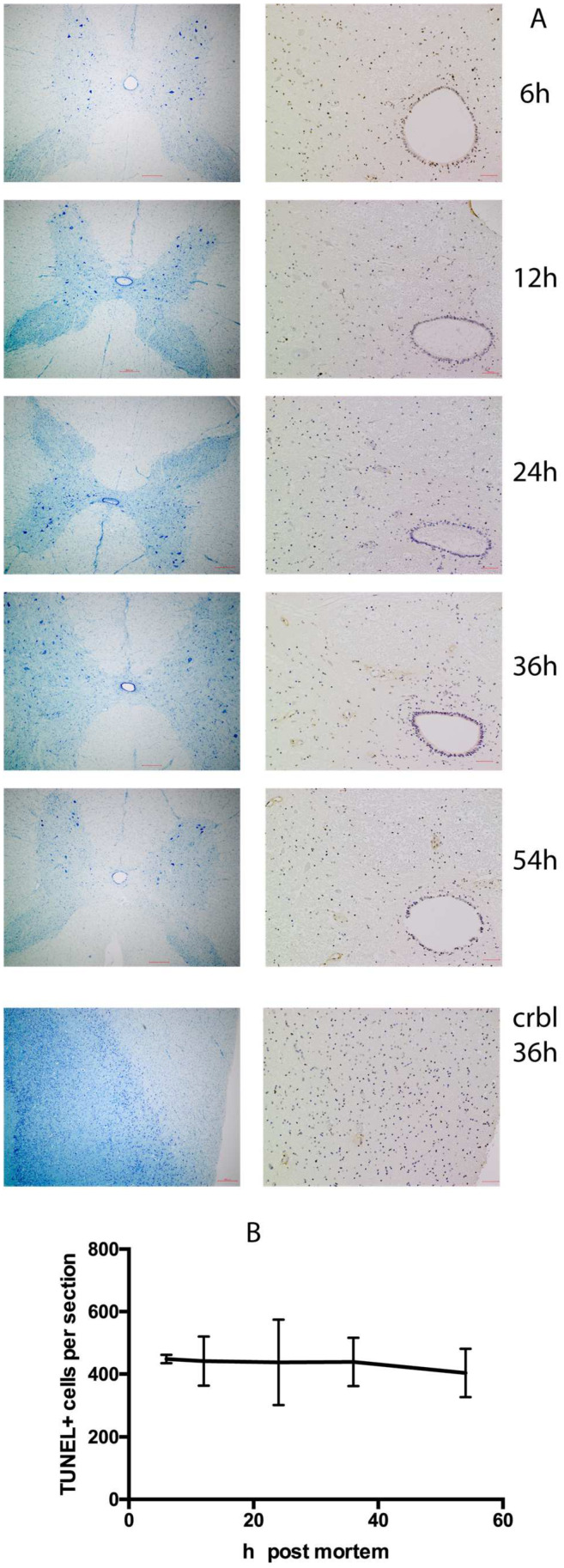
Changes in apoptotic cell number with time postmortem. (**A**) Pairwise staining with Toluidine Blue (left) and TUNEL (right) of the transections of the goat spinal cord at different time (h) postmortem. Lower pair of images is the sample of cerebellum from the same animal at 36 h postmortem. Scale bars 300 µm on all images. (**B**) Number of TUNEL-positive cells per spinal cord trans-section (*n* = 3 for each time point). Values are the mean ± SD; no difference at *p* < 0.1 between any pair.

TUNEL staining of spinal cord samples over 6–54 h postmortem revealed no difference in the number of positive cells per cross-section. Many TUNEL-positive cells were located in the gray commissure around the central canal of the spinal cord; no increase in TUNEL-positive cells was recorded in either the posterior or anterior horns of the gray matter. In comparison, cerebellum tissue collected at the 36 h time point had 2–3 times more TUNEL-positive cells per field of view (Figure 1).

**Figure 2:**
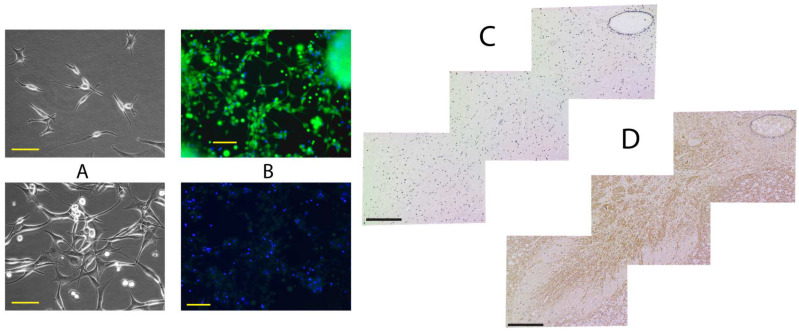
Differentiation, location and survival of neuronal cells. Cells plated after magnetic separation in 24 h ((**A**) upper) and after 20 days/passage 3 ((**A**) lower), scale bar 100 µm. Up to 80% of the cells in Neuronal differentiation medium stain positive with anti-Neurofilament-AlexaFluor488 antibodies ((**B**) upper); anti-Cytokeratin 5 AlexaFluor488 antibodies as isotype control ((**B**) lower), scale bar 100 µm. Reconstruction from 3 neighboring deparaffinated sections of postmortal spinal cord stained with TUNEL (**C**) and antibodies against GD2 (**D**), scale bar 200 µm.

Magnetically enriched CD24-positive cells were able to attach on poly-ornithine-covered flasks and grow undifferentiated at least up to 3 weeks (Figure 2A). When incubated in neuronal differentiation medium, cells became about 80% positive when stained with fluorescently labelled antibodies against the neurofilament (Figure 2B), suggesting that the harvested cells were predominantly neuronal progenitor cells capable of differentiating into neurons.

The staining of fixed and deparaffinated spinal cord cross-sections with antibodies against oligosaccharide determinant of disialoganglioside GD2 demonstrated that GD2, commonly associated among others with neural progenitor cells, prominently stains anterior horns of gray matter (Figure 2D). The sections neighboring to GD2-stained were visualized by the TUNEL method, which did not reveal any changes in staining density at GD2-positive areas (Figure 2C).

### 2.2. Western Blot Analysis

**Figure 3:**
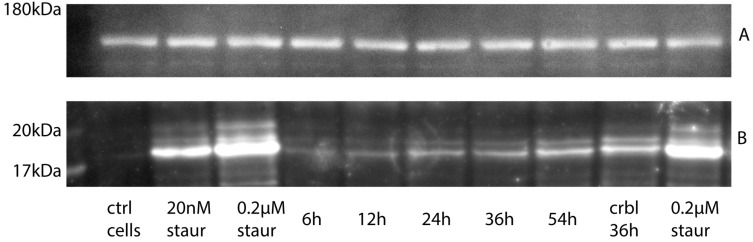
Western blot of postmortal tissues and cultured cells probed with antibodies against active caspase 3. Visualization with antibodies against Neurofilaments as loading control (**A**) and active caspase 3 (**B**). Lyzed cultured neuronal cells treated with 20 nM–0.2 µM staurosporine used as positive control, untreated cultured neuronal cells used as negative control. Gel images were cropped to areas with expected target molecular mass to clarify the representation.

Despite no difference in the number of TUNEL-positive cells, the amount of cleaved active caspase 3 as measured by Western blot at the same time intervals was moderately increasing over time (Figure 3).

### 2.3. Cytofluorometric Analysis

**Figure 4:**
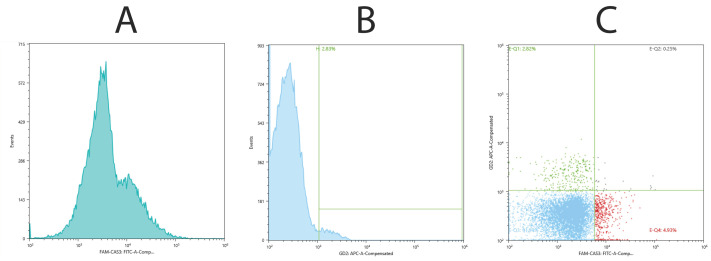
Survival of neuronal cells harvested from 24 h postmortal spinal cord and enriched by magnetic beads with CD24 antibodies. Cells were stained with FAM-VAD-FMK and GD2-APC and analyzed by flow cytometry. Fluorescence distribution histogram from FAM-VAD-FMK channel (**A**) and GD2-APC channel (**B**); two-dimensional graph of both channels (**C**).

Magnetically enriched CD24-positive cells were stained with FAM-VAD-FMK and antibodies against GD2 conjugated to APC. About 2.8% of the counted cells were GD2-positive. The GD2-positive population did not overlap much with cells having high pan-caspase activity measured with FAM-VAD-FMK (Figure 4A–C).

## 3. Discussion

We utilized the magnetic beads binding CD24 on cell surfaces for enrichment of the neuronal cells from cadaveric spinal cords. However, CD24 is not strictly specific or restricted to neuronal cell lineages. Other cells such as adipocyte progenitors [16], proximal tubule progenitors from the nephron [17], renal progenitors [18], pancreatic progenitors [19], and a number of cancer stem cells [20] can expose this antigen on their surface. We did not expect most of those cell types in the environment of the adult spinal cord, so the choice of CD24 for neuronal cell enrichment seemed rational. It is also known that CD24+ cells have greater potential than CD24− cells to differentiate into cells of the neural lineage [21], while surface CD24 antigen expression in neural stem cells is lower than in mature neuroblasts and neurons [22]. In our study, the cells positively selected from mildly digested cadaveric spinal cords by CD24 magnetic beads retained high viability and low caspase activity (~90% FAM-VAD-FMK-negative cells). A slow increase was observed in the amount of activated caspase 3 measured by Western blot in the spinal cord samples over time postmortem, coinciding with no increase in the number of the apoptotic cells measured by TUNEL staining. This could be explained by the hypothesis that, initially, apoptotic cells slowly continue the process of caspase activation under static cold storage conditions, while no new apoptotic cells formed postmortem.

Fascinating studies of postmortal gene transcripts have been performed on mice brain and liver tissues analyzed during 0–48 h postmortem time points. Among other changes, the authors revealed a number of pro- and anti-apoptosis genes inter-regulating each another. Moreover, the transcripts of the following notional cancer genes were significantly increased: Rbl1, Bcl2l11, Map3k2, Bcl6, Ppp1, Cdc42, and Ppp2. Most of those discovered genes with elevated expression levels play important roles in the survival of stem cells as well [23]. In its modern form, apoptosis seems to have evolved to keep homeostasis in living multicellular organisms and their development [24]. However, when death arises, there is no need for surviving cells to sacrifice themselves for the sake of the integrated organism anymore. So, as a meaningless practice, apoptosis in a dead body may be abandoned, and an egoistic principle “each cell to their own” could be applied instead, favoring long-term postmortal survival of some cell populations. When such cells are reintroduced into a new functioning organism, we would expect switching their modus operandi back to apoptosis-governed cellular behavior.

To characterize separated neuronal cells as progenitors and localize the neural progenitors on histological samples, we employed techniques for the detection of the ganglioside GD2 and differentiation of harvested cells in neuronal differentiation medium. It was reported that upon differentiation of human embryonic stem cells into neural progenitors, the glycosylation of prominent glycolipids is switched from globo- and lacto-series to mostly ganglio-series, in this case dominated by GD3 [25], which is the precursor for GD2 processed further with GM2/GD2 synthase, i.e., the enzyme which converts ganglioside GD3 to GD2 [26]. The majority of GD2+ cells are also CD44 and CD24 positive and previously considered to associate with cancer stem cell surface phenotypes. Furthermore, GD3 synthase knockdown completely abrogated tumor formation in vivo [27,28]. We found that ~2.8% of CD24-enriched cells from the cadaveric spinal cord were GD2-positive. Since histological staining attributes those cells mainly to anterior horns, further development of preclearing methods and their validation will be necessary to improve yield and specificity of neural progenitor isolation.

Our animal model has substantial differences with humans when comes to genetical, behavioral or physiological features. Yet, we see our choice of goats advantageous over rodent models when considering similarities in organ size, metabolic rate, lifespan, and clinical procedures [10]. Although genome sequences and gene manipulation tools are now available for goats, there are no gene expression validation panels available for characterizing stem cells in this animal. Although our knowledge of the processes responsible for pluripotency maintenance is inadequate [29], the validation of committed neuronal progenitor cells was possible via glycolipid (not species-specific) GD2 antigen expression. From experimental point of view, the harvesting of fetal material could be advantageous as they would have higher replicative potential; however, such technology will not be possible for transfer to future human use.

Harvesting stem cells from cadavers would be a more advantageous practice compared to harvesting whole organs due to the resilience of separated cells to cryopreservation. The best-known clusters of NSCs are located in the neurogenic areas of the subventricular zone of the lateral ventricles and the subgranular zone of the dentate gyrus [30] which would require harvesting of the postmortem brains; it is expected that this would cause considerable ethical controversy. On the other hand, the spinal cord is not an ethically stigmatized location.

## 4. Materials and Methods

### 4.1. Tissue Sampling

Goats (3.4–4.8 years old, 45–51 kg, females) were slaughtered for food production outside of the university. Spines and cerebella were purchased (from Nisshin Seifun Group, Tokyo, Japan), then collected and transported at +4 °C to the laboratory. Samples of the spinal cords were harvested at T4, T7, T10, T12, and L2 levels at 6, 12, 24, 36, and 54 h postmortem; between sampling, spines were kept in refrigerator at +4 °C and 100% humidity.

### 4.2. Histological Analysis

Since the effect of postmortem delay and fixation time are crucial preanalytical variables that can influence genomic analysis and immunohistochemistry outcomes [31], the cross-section segments of the spinal cords between dorsal/ventral roots with thicknesses of 4 mm were excised and immediately fixed with 4% paraformaldehyde in PBS (FUJIFILM Wako, Osaka, Japan) for 30 min at 37 °C. The fixed tissue were kept in 0.5% paraformaldehyde in PBS at +4 °C until they were shipped for processing in NHSL:New Histo Science Laboratory Co. (Tokyo, Japan). TdT-mediated dUTP nick end Labeling staining was performed using an In Situ Cell Death Detection Kit (Roche, Basel, Switzerland). A total of three cross-sections per time point were analyzed; the primary anti-GD2 antibody (14G2a, Santa Cruz Biotechnology, Dallas, TX, USA) was visualized by peroxidase reaction with DAB of secondary antibodies conjugate; and toluidine blue (TB) stains were performed in a processing lab. Images were taken with a BZ-9000 BioRevo microscope (Keyence, Osaka, Japan) equipped with 4× and 20× objectives.

The open-source image processing package Fiji (distribution of ImageJ2 Release 2.9.0) was used to calculate TUNEL-positive cells; GraphPad Prism (ver. 5.0 f for Mac) was used for statistical calculation of TUNEL-positive cells and its graphical representation. Statistical analyses were performed using Student’s *t* test. Data are shown as mean ± SD values. *p*-values > 0.1 were considered not statistically significant.

### 4.3. Cell Harvesting and Enrichment

Neural Dissociation System 6 (CHI Scientific, Maynard, MA, USA) was used to enzymatically disassemble the spinal cord samples. The resulting cells were washed twice with 0.2 µm filtered solution containing phosphate-buffered saline (PBS), pH 7.2, 0.5% bovine serum albumin (BSA), and 2 mM EDTA. The cells were positively selected using four LS separation columns with a CD24 MicroBead Kit on QuadroMACS™ Separator (all from Miltenyi Biotec, Bergisch Gladbach, Germany).

### 4.4. Western Blot Analysis

Small pieces of gray matter, 70–100 mg in total (wet mass), were dissolved in 2× Laemmli Sample Buffer and applied on 4–15% precast polyacrylamide gel (Criterion TGX), together with prestained Protein ladder EXtended PS13 (5–245 kDa, GeneON, Ludwigshafen, Germany) and positive control samples (cultured primary neurons from adult goat spines treated with the apoptosis-inducing agent staurosporine). Separation was performed in Criterion™ Vertical Electrophoresis Cell (BioRad, Hercules, CA, USA) with a constant voltage 140 V. Proteins from the gel were transferred onto Immobilon-FL PVDF membranes (0.45 µm, Millipore now Merck KGaA, Darmstadt, Germany) blocked with Pierce Protein-Free Blocking Buffer (Thermo Scientific, Waltham, MA, USA), cut on the level of 75 kD standards lines and stained separately with primary antibodies against active caspase 3 (ab214430, AbCam, Cambridge, UK) and neurofilaments (anti-Neurofilament M antibody, Merck/Chemicon, Darmstadt, Germany) as loading control. They were then developed with Amersham ECL Prime Reagent and visualized from contact-exposed X-ray film. Only areas of interest were manually scanned (with GT-X980 scanner, Epson, Suwa, Nagano, Japan) from the films due to constraints of file size and optical density processing.

### 4.5. Differentiation

Enriched CD24-positive cells were seeded at a density of 4 × 10^5^ cell/mL on dishes covered with poly-L-Ornithine solution (0.01%, Sigma/Merck, Darmstadt, Germany) in STEMdiff (Stemcell Technologies, Vancouver, BC, Canada) media, and parts of cell batches were incubated in neuronal differentiation medium (ENStem-A™, Merck, Darmstadt, Germany) for 240 h. The cells were stained with DAPI, and the neurons were visualized with anti-Neurofilament H antibody (clone NE14, Alexa Fluor 488 conjugated, Merck) after fixation and permeabilization (FIX & PERM Cell Permeabilization Kit, Invitrogen/Thermo Scientific, Waltham, MA, USA). Anti-Cytokeratin 5,6 Antibodies (clone D5/16B4, Alexa Fluor 488 conjugated, Merck) were used as isotype/dye negative control since cytokeratins are intermediate filaments not normally expressed in neurons.

### 4.6. Cytofluorometric Analysis

Enriched CD24-positive cells (~10^7^) were simultaneously stained with Vybrant™ FLICA pan-Caspase Apoptosis Assay Kits for flow cytometry (Invitrogen/Thermo Scientific, Waltham, MA, USA) 1:150 and APC conjugated anti-human Ganglioside GD2 Antibody (BioLegend, San Diego, CA, USA) 1:100 in 0.25 mL of IsoFlow Sheath Fluid (Beckman Coulter, Pasadena, CA, USA) for 30 min at 37 °C. Then the cells were washed once with warm IsoFlow fluid and measured by a Sony SH800Z Sorting Flow Cytometer (Sony Biotechnology, Tokyo, Japan) with automatic channel compensation.

## 5. Conclusions

Data from this study together with our earlier report on the long-term survival of neural progenitors in human-sized mammals [32] suggest postmortal spinal cords as a valuable source for harvesting allogenic neural progenitor cells. Apoptotic cell death events are relatively rare in postmortal spinal cords and are not increased in areas of neural progenitor cell’s location, within the measured postmortal intervals, or among the CD24/GD2-positive cells.

## Data Availability

Raw images and flow cytometry files are available upon request from the corresponding author.

## Abbreviations

APC	allophycocyanin
CD24	sialoglycoprotein antigen
CSCs	cadaveric stem cells
GD2	disialoganglioside antigen
EDTA	ethylenediaminetetraacetic acid
FAM-VAD-FMK	fluorescent cell-permeable polycaspase inhibitor
FLICA	fluorochrome-labeled inhibitors of caspases
MSCs	mesenchymal stem cells
NSCs	neural stem cells
PVDF	polyvinylidene fluoride
PBS	phosphate buffer saline
TUNEL	terminal deoxynucleotidyl transferase mediated dUTP Nick End Labeling assay
